# Metformin combined with rapamycin ameliorates podocyte injury in idiopathic membranous nephropathy through the AMPK/mTOR signaling pathway

**DOI:** 10.1007/s12079-023-00781-8

**Published:** 2023-09-13

**Authors:** Meichen Ma, Yue Pan, Yue Zhang, Mei Yang, Ying Xi, Baoxu Lin, Wudi Hao, Jianhua Liu, Lina Wu, Yong Liu, Xiaosong Qin

**Affiliations:** Department of Laboratory Medicine, Shengjing Hospital of China Medical University, No. 36 Sanhao Street, Heping District, Shenyang, 110004 People’s Republic of China

**Keywords:** Idiopathic membranous nephropathy, Metformin, Rapamycin, AMPK/mTOR

## Abstract

Autophagy activation protects against podocyte injury in idiopathic membranous nephropathy (IMN). The AMPK/mTOR signaling pathway is a vital autophagy regulatory pathway. Metformin promotes autophagy, whereas rapamycin is an autophagy agonist. However, the therapeutic mechanisms of metformin and rapamycin in IMN remain unclear. Thus, we examined the mechanisms of action of metformin and rapamycin in IMN by regulating the AMPK/mTOR autophagy signaling pathway. Female Sprague–Dawley (SD) rats were treated with cationic bovine serum albumin (C-BSA) to establish an IMN model and were randomly divided into IMN model, metformin, rapamycin, and metformin + rapamycin groups. A control group was also established. Metformin and rapamycin were used as treatments. Renal histological changes, urinary protein excretion, the protein expression levels of key AMPK/mTOR signaling pathway proteins, renal tissue cell apoptosis, and autophagy-associated proteins (Beclin 1 and LC3) were examined. In addition, a C5b-9 sublysis model using the MPC-5 mouse podocyte cell line was established to verify the effect of metformin combined with rapamycin on podocytes. Metformin combined with rapamycin improved urinary protein excretion in IMN rats. Metformin combined with rapamycin attenuated the inflammatory response, renal fibrosis, and podocyte foot process fusion. In addition, it improved autophagy in podocytes as demonstrated by the enhanced expression of Beclin-1, p-AMPK/AMPK, LC3-II/I, and autophagosomes in podocytes and decreased p-mTOR/mTOR expression. In conclusion, metformin combined with rapamycin decreased proteinuria, improved renal fibrosis and podocyte autophagy via AMPK/mTOR pathway in IMN rats.

**Graphical Abstract:**

The metformin and rapamycin decreased proteinuria and inproved renal fibrosis in IMN model rats.
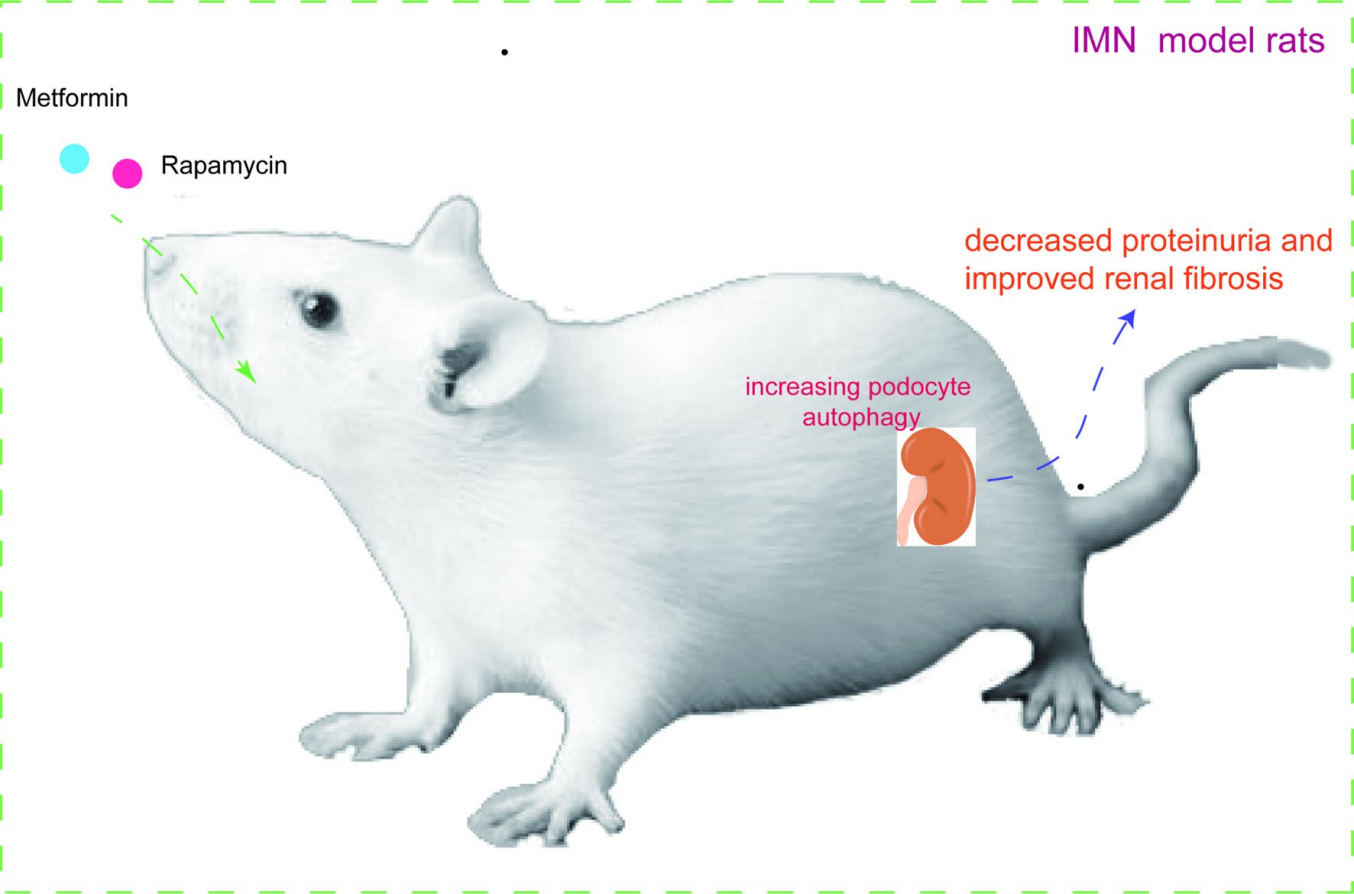

**Supplementary Information:**

The online version contains supplementary material available at 10.1007/s12079-023-00781-8.

## Introduction

Idiopathic membranous nephropathy (IMN), a common kidney disease, causes adult nephrotic syndrome (Alsharhan and Beck 2021; Cattran and Brenchley 2017). It is characterized by the expansion of subepithelial immune complexes (e.g., immunoglobulin [IgG] and complement components [C5b-9 and C3]) and circulating autoantibodies in the kidneys (Ronco and Debiec 2015). Podocytes play an important role in the glomerular filtration barrier of the kidneys (Sha et al. 2015). Autophagy in podocytes helps maintain cell homeostasis via protein and organelle degradation, and autophagy dysfunction is known to contribute to podocyte injury (Mizushima et al. 2008).

Until now, IMN treatments have included glucocorticoids, supportive care, and cytotoxic or immunosuppressive agents. Patients with MN must receive optimum supportive care (Beck et al. 2013; Tian et al. 2019). Clinicians often find that the early initiation of glucocorticoids, immunosuppressive agents, and glucocorticoids can be harmful to patients. In addition, immunosuppressive or cytotoxic agents, which frequently exhibit evident negative effects, do not always have the desired treatment effects in patients with MN. Thus, identifying effective medical treatments is important for improving the treatment strategies for patients with IMN.

Metformin is widely used for diabetes treatment (Aroda et al. 2017; Barzilai et al. 2016). Metformin has antitumor, anti-inflammatory, and anti-aging activities, indicating that it can effectively treat other diseases (Cameron et al. 2016; Cuyàs et al. 2018). Although numerous aspects of metformin have been investigated, the anti-inflammatory effects of metformin on tumor cell inhibition (Feng et al. 2014), treatment of kidney and liver diseases, anti-aging effects, and apoptosis metabolism have been mostly emphasized (Crowley et al. 2017). In addition, metformin was found to attenuate cartilage degeneration in an experimental osteoarthritis model through AMPK/mTOR regulation (Feng et al. 2020). Metformin also enhances autophagy in diabetic kidney disease via AMPK/SIRT1-FoxO1 pathway (Ren et al. 2020). However, it is unclear if metformin regulates AMPK/mTOR in the development and progression of IMN.

AMPK activation by metformin indicates that the metformin-induced increase in metabolic profiles is associated with the induction of autophagy through AMPK activation (El-Arabey 2017). Interestingly, the immunosuppressant rapamycin causes mTOR inhibition (Wang et al. 2017). Rapamycin can effectively inhibit mTOR/P70S6K/4EBP1 signal pathway, activate podocyte autophagy to reduce podocyte apoptosis (Jin et al. 2018). Therefore, we explored if the combination of rapamycin and metformin could play a vital role in the autophagy of IMN. Based on the AMPK/mTOR signaling pathway, we investigated if metformin combined with rapamycin could activate autophagy to improve pathological changes in IMN model rats, including inflammatory infiltration of macrophages, secretion of inflammatory factors, and renal fibrosis. We selected the MPC-5 mouse podocyte cell line for in vitro experimental to verify the effect of metformin combined with rapamycin on podocytes. With this combined approach, we aim to shed light on the treatment of IMN potential of metformin combined with rapamycin and study the possible underlying mechanisms of anti-inflammatory, renal fibrosis, and podocyte foot process fusion.

## Materials and methods

### Animal preparation

Fifty Sprague–Dawley (SD) rats (female; 160–180 g; 10 weeks old) were collected from the Charles River, Beijing, China, and reared in the animal laboratory of our hospital. The rats were placed in a 12:12 h light/dark environment and provided with sufficient water and food. The rats were divided into control (C), IMN model, metformin, rapamycin, and metformin + rapamycin groups (n = 10 rats/group). The experimental protocol was approved by the Ethics Committee of Shengjing Hospital of China Medical University (2022PS092K).

### Rat model establishment and treatment

The rats in the IMN model group received cationic bovine serum albumin (C-BSA; 9058; Chondrex, Redmond, USA) injected through the tail vein (6.5 mg·kg^−1^) 15 times in 30 days. Rats in the Control group were injected with sterile saline solution (6.5 mg·kg^−1^) according to the same schedule. To ensure the success of the IMN model rats, 24-h proteinuria was examined. The successful IMN rats were randomly allocated to four groups: IMN model, metformin, rapamycin, and metformin + rapamycin. Rapamycin and metformin groups were pretreated with C-BSA. Metformin was administered daily by oral gavage (200 mg/kg·d), and rapamycin was administered via intraperitoneal injection at 1.5 mg/kg·d. The IMN model groups were offered distilled water solutions. All rats were sacrificed after the four-week treatment, and blood samples were collected. In addition, kidney samples were removed and stored (Supplementary Fig. S1).

### Serum and urine collection and analysis

Metabolic cages were used to collect urine samples at 24 h. Protein concentration was quantified using the Bradford method. Blood samples were collected from the abdominal aorta and centrifuged again. Serum samples were kept at − 80 °C. Urine protein levels were measured using an automatic biochemical analyzer.

### qRT-PCR

RNA extraction kit procured from Biomed, China, was used to obtain total RNA samples from renal tissue, followed by quantification using Nanovue spectrophotometer. Further, reverse transcription of RNA for complementary DNA (cDNA) in qRT-PCR (make of Applied Biosystems, USA) with GAPDH as an internal control, was performed via First-Stand cDNA Synthesis SuperMix and Trans Start Green qPCR SuperMix procured from TransGen Biotech, China. Primers used are: TGF-β (F: 5′-AGCAACAATTCCTGGCGATACCTC-3′, R: 5′-TCTTCAGCTTTCCAGCGGAC-3′), α-SMA (F: 5′-TGCTGGACTCTGGAGATGGTGTG-3′, R: 5′-CGGCAGTAGTCACGAAGGAATAGC-3′), GAPDH (F: 5′-ACGGCAAGTTCAACGGCACAG-3′, R: 5′-CGACATACTCAGCACCAGCATCAC-3′). The 2^−ΔΔCt^ method (Livak and Schmittgen 2001) was employed for estimating the expression levels of relative mRNA in all assays, which were done in triplicate.

### ELISA

Blood samples were collected from five rats and serum samples were subsequently obtained. VEGF, TNF-α, and IL-6 levels in the serum and MPC-5 cells were determined using ELISA according to the manufacturer's protocol (BD Biosciences, USA).

### Histological examination

The collected kidney specimens were fixed using buffered 4% paraformaldehyde, embedded in paraffin, sectioned into slices (4-μm thick), and stained with hematoxylin and eosin (HE) following the manufacturer’s instructions.

### Transmission electron microscopy (TEM)

The samples (Renal tissues and podocytes cells) were fixed in 2.5% glutaraldehyde in 0.1 M sodium cacodylate buffer (pH 7.4) and post-fixed in 2% osmium tetroxide. Subsequently, they were embedded in epoxy resin. Sections (0.1-μm thick) were stained with lead citrate and uranyl acetate. Representative autophagic vacuoles in podocytes observed using TEM. Transmission electron micrographs were obtained at 60 kV using a Zeiss EM-10 electron microscope (Zeiss, Gottingen, Germany).

### Immunohistochemical assay

After deparaffinization and rehydration, the renal tissue slices were treated with H_2_O_2_ (3%) in methanol for 30 min and blocked with 5% fat-free milk for 1 h. The slices were then incubated with the primary antibody (anti-CD68, ab283654, 1:100, Abcam, UK) at 4 °C for 24 h. Bound antibodies were identified with a biotin-labeled secondary antibody using the ABC kit, visualized with diaminobenzidine, and analyzed using light microscopy after washing (Thermo Fisher Scientific, Waltham, USA). The immunoassayed slides were scored thrice and the mean values were calculated.

### Western blot

Tissues were collected and the total protein concentration was examined using Coomassie brilliant blue. Total protein was separated using 10% SDS-PAGE gel electrophoresis and transferred onto a polyvinylidene fluoride or polyvinylidene difluoride (PVDF) membrane. PVDF membranes were blocked in skimmed milk (5%) in PBS at 25 °C for 2 h before incubation with primary antibodies (AMPK, mTOR, p-AMPK, LC3, p-mTOR, Beclin 1, ATG5, ATG7, ATG12, and β-actin) at 4 °C for 24 h. The PVDF membranes were incubated with anti-rabbit IgG (1:2000, A6154MSDS, Sigma-Aldrich, St. Louis, USA) for 2 h at 2 °C. Protein bands were visualized and analyzed using an ECL system (Thermo, Waltham, USA) and the ImageJ software, respectively.

#### Podocyte culture and treatment

The MPC-5 cell line (conditionally immortalized) was purchased from ATCC (Manassas, VA, USA). MPC-5 cells were kept in RPMI 1640 supplemented with 10% heat-inactivated fetal calf serum (Thermo Fisher Scientific, Waltham, USA), 100 U ml^−1^ penicillin, and 100 μg ml^−1^ streptomycin and grown at 33 °C for proliferation.

To establish the model of C5b-9 sub lysis of MPC-5 podocytes, 0.15 mol/L sodium chloride with 1% yeast polysaccharide was prepared, boiled in double-distilled water for 1 h, cooled to 25 °C, and centrifuged at 4000 rpm for 30 min. The supernatant was discarded and the precipitate was used as standby. As previously described (Ishikawa et al. 1993; Liu et al. 2017), cells were cultivated in medium containing 10% Yeast polysaccharide-activated serum (zymosan-activated serum; ZAS) at 37 °C for 60 min. The lactate dehydrogenase release rate of the culture medium supernatant was determined based on the manufacturer’s protocol to determine the optimal level of ZAS for the establishment of the podocyte C5b-9 sublysis model. MPC-5 cells were cultivated in 5% CO_2_ at 37 °C for 14 days in DMEM and then exposed to metformin (10 μmol/L) or rapamycin at a dose of 20 nmol/L for 24 h. The levels of TNF-a (A), IL-6 (B), and VEGF (C) in the MPC-5 cell supernatant measured using ELISA.

#### F-actin staining

Podocytes were fixed using 4% paraformaldehyde, permeabilized for 10 min on ice using TritonX-100 (0.1%) (T8787, Sigma, USA), and incubated with rhodamine-phalloidin (ab235138, Abcam, Cambridge, UK) (1:100) for 1 h at 25 °C. A confocal microscope (20 × , LSM 780, Zeiss) was used to examine the cells.

#### Statistical evaluation

Data were examined using SPSS 22.0 and GraphPadPrism7 and displayed as means ± SD (standard deviation). The experiment was repeated three times. Repeated-measures ANOVA (one-way analysis of variance) and Tukey’s test were used to identify significant differences. Differences were considered statistically significant at *P* < 0.05.

## Results

### Metformin combined with rapamycin reduced proteinuria and renal fibrosis in C-BSA-induced IMN rats

**Fig. 1:**
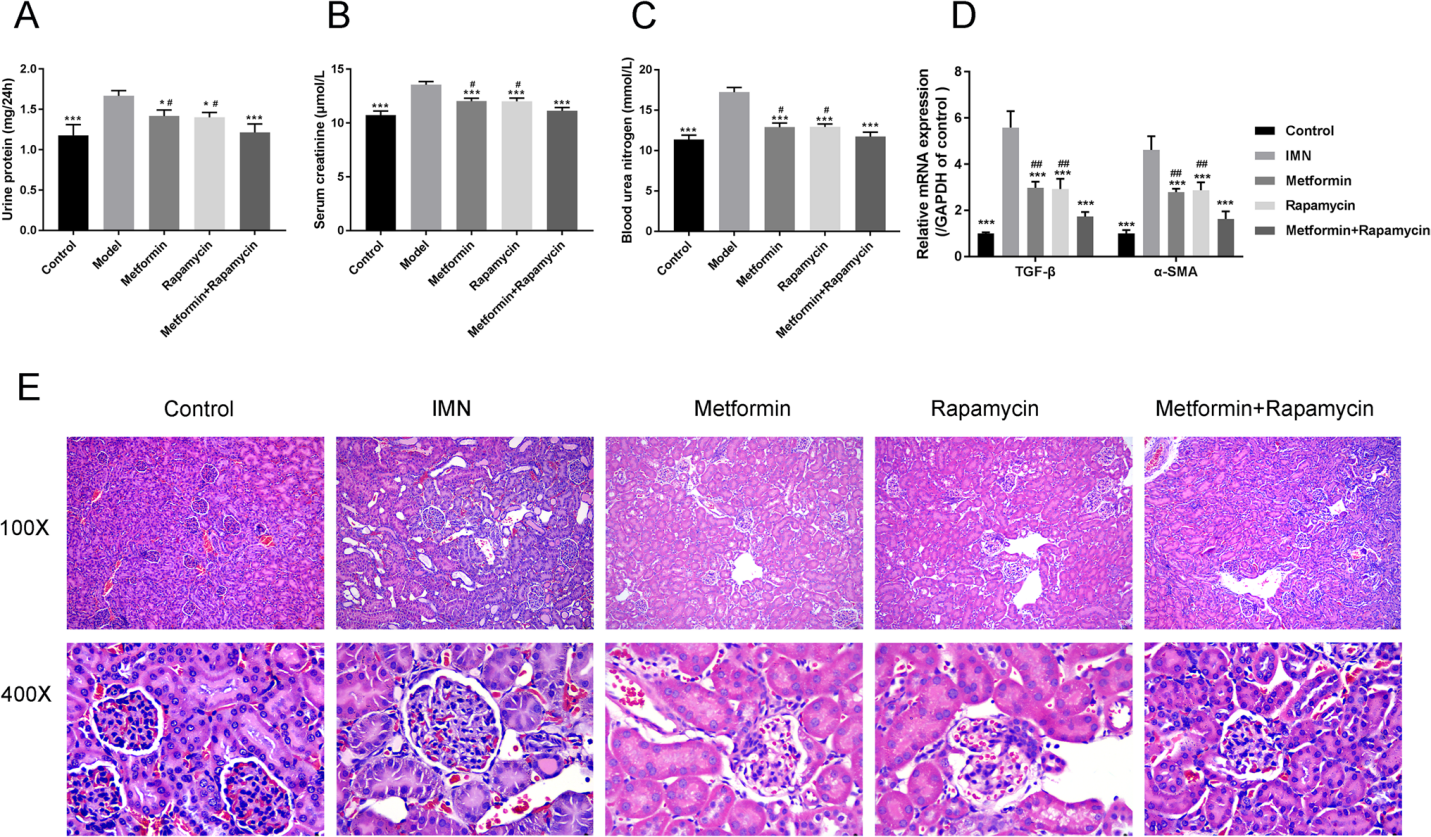
Metformin combined with rapamycin treatment reduced proteinuria and glomerular pathomorphology in C-BSA-induced IMN rats. **A** Twenty-four-hour urinary protein quantity (mg/24 h) of each group after administration of metformin, rapamycin, and metformin + rapamycin. **B** Serum creatinine levels (μmol/L) of all groups. **C** Blood urea nitrogen (BUN) levels (mmol/L) of all groups. **D** The expression of TGF-β and α-SMA in the control group, IMN group, metformin, rapamycin, and metformin + rapamycin groups. The relative expression is homogenized when statistics are performed, and the sham group is defined as 1.** E** Morphological changes in hematoxylin and eosin (HE)-stained rat renal tissue sections using light microscopy (Magnification 100 × , 400 ×). *P < 0.05, **P < 0.01, ***P < 0.001 vs. IMN group; ^#^*P* < 0.05 vs. metformin + rapamycin group

The 24-h urinary protein, blood urea nitrogen (BUN), and serum creatinine levels significantly increased in C-BSA-induced IMN model rats compared with those of C group rats (*P* < 0.001) (Fig. 1A–C). The levels of these indicators were significantly reduced by metformin, rapamycin, and metformin + rapamycin (*P* < 0.05, *P* < 0.01, and *P* < 0.001, respectively) compared with those of the model group after four weeks of metformin and rapamycin administration. Interestingly, this effect was significantly enhanced by metformin + rapamycin (*P* < 0.05). qRT-PCR showed that the mRNA expression of the key renal fibrosis factors TGF-β and α-SMA was significantly higher in the IMN group than in the control group, while the expression of TGF-β and α-SMA was decreased in the metformin, rapamycin, and metformin + rapamycin groups (*P* < 0.01, and *P* < 0.001, Fig. 1D).

HE-stained tissues were observed using light microscopy to examine kidney morphology. The model rats showed increased renal glomeruli, capillary stenosis, and thickened capillary walls with scattered subepithelial basement membrane projections. The metformin, rapamycin, and metformin + rapamycin groups exhibited better morphological alterations compared with those in the model group (Fig. 1E). Our data indicate that metformin + rapamycin treatment reduced C-BSA-induced proteinuria, and renal fibrosis in IMN rats.

### Metformin combined with rapamycin reduced the inflammatory response in C-BSA-induced IMN rats

**Fig. 2:**
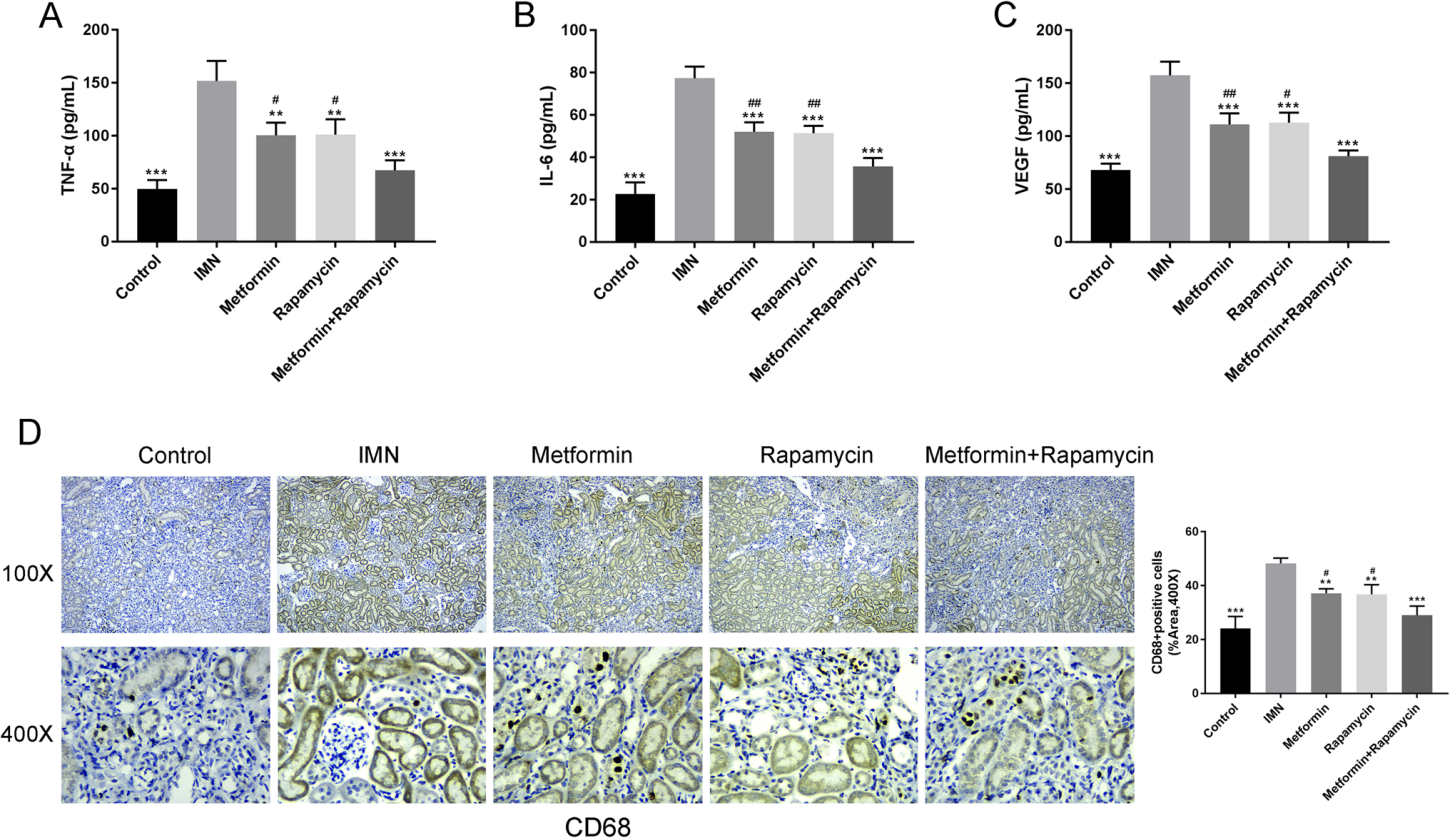
Metformin combined with rapamycin reduced the inflammatory response in cationic bovine serum albumin (C-BSA)-induced IMN rats. **A–C** Determination of the TNF-a (**A**), IL-6 (**B**), and VEGF (**C**) levels in each group after immunization using ELISA. **D** The glomerular macrophage marker CD68 detected using immunohistochemistry. Bar diagrams show quantitation of IHC analysis. **P < 0.01, ***P < 0.001 vs. IMN group. ^#^*P* < 0.05, ^##^*P* < 0.01 vs. metformin + rapamycin group

Serum IL-6, TNF-α, and VEGF levels were evaluated using ELISA. The concentrations of inflammatory cytokines VEGF, TNF-α, and IL-6, implicated in the innate immune response, were significantly higher in IMN rats compared with those in C group rats (*P* < 0.001). Treatment with metformin, rapamycin, and metformin + rapamycin decreased the levels of TNF-α, VEGF, and IL-6 (*P* < 0.01, *P* < 0.001, Fig. 2A-C). Moreover, combination treatment significantly reduced TNF-α, IL-6, and VEGF levels compared to metformin or rapamycin treatment alone (*P* < 0.05; *P* < 0.01), indicating the synergistic action of metformin and rapamycin. Furthermore, CD68, the marker used to identify glomerular macrophages, was detected using immunohistochemistry (*P* < 0.05, *P* < 0.01, *P* < 0.001, Fig. 2D). The number of brown CD68-positive cells in renal tissue was increased in IMN rats. However, metformin, rapamycin, and metformin + rapamycin treatments decreased the number of CD68-positive cells, suggesting that metformin combined with rapamycin could reduce the infiltration degree of macrophages.

### AMPK/mTOR signaling is responsible for the effect of metformin + rapamycin on IMN rats

**Fig. 3:**
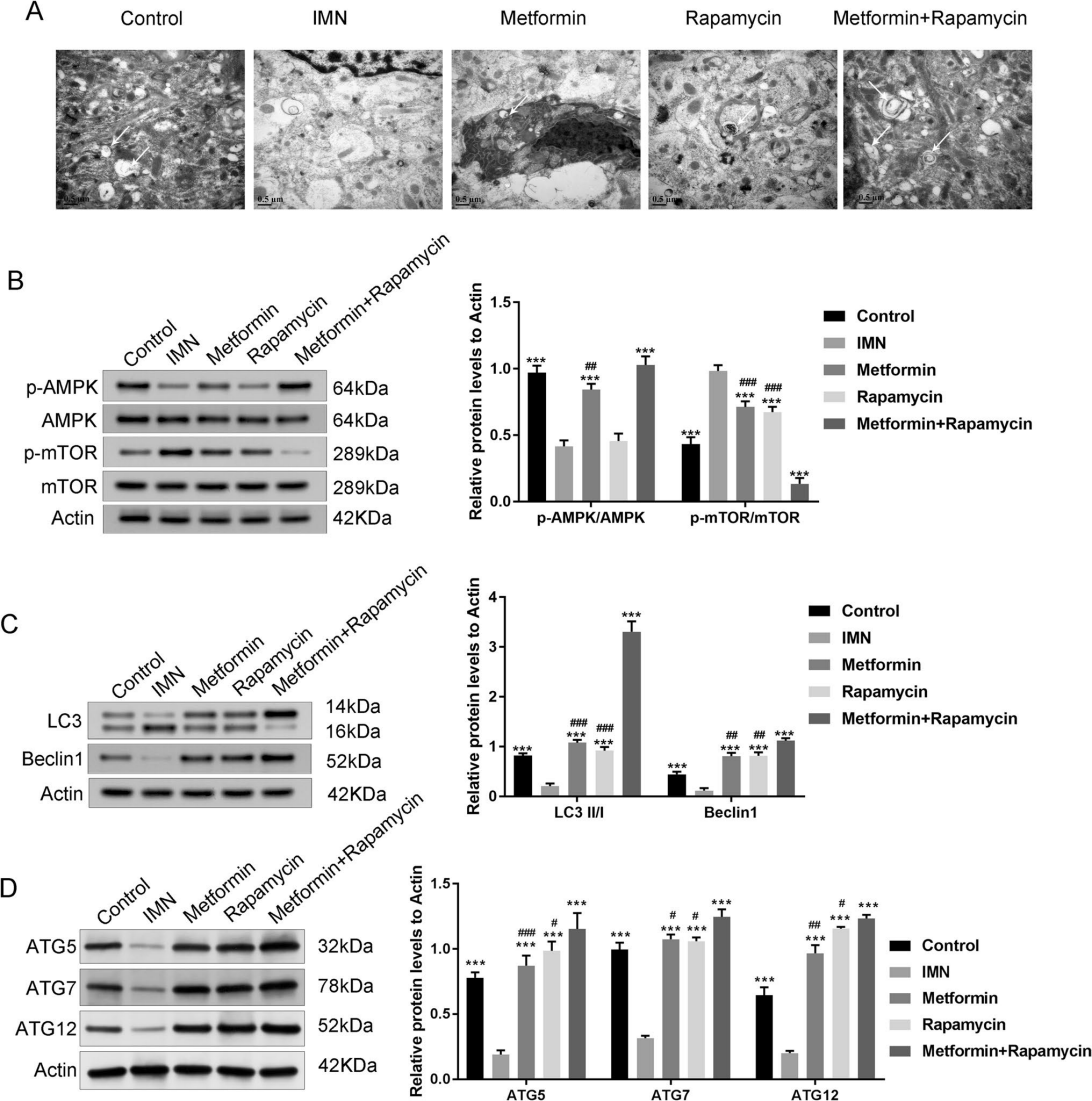
Metformin combined with rapamycin affected the expression of AMPK/mTOR-related pathway proteins and promoted autophagy. **A**Transmission electron microscopy (TEM) micrograph displaying the ultrastructure and the podocyte changes of the kidneys in the tested groups (Scale bars = 0.5 μm). **B** Western blot analysis of the expression levels of AMPK/mTOR signaling pathway proteins (p-mTOR, mTOR p-AMPK, and AMPK). **C** Western blot analysis of the expression levels of autophagy markers (LC3I, LC3II, and Beclin1). **D** Western blot analysis of the protein expression levels of autophagy markers (ATG5, ATG7, and ATG12). The experiment was repeated three times, and the data are represented as the mean ± SD. ***P < 0.001 vs. IMN group; ^#^*P* < 0.05, ^##^*P* < 0.01, ^###^*P* < 0.001 vs. metformin + rapamycin group

TEM revealed clear morphological changes in autophagosomes in podocytes of renal tissues (Fig. 3A). In the model group, C-BSA injection decreased the number of autophagosomes in podocytes. However, treatment with metformin and rapamycin increased the number of autophagosomes in podocytes. The effect of metformin + rapamycin on the AMPK/mTOR signaling pathway and apoptotic proteins in the kidney tissue of rats was observed. The protein expression ratio of p-AMPK/AMPK in the model rats was lower than that in the C group. Furthermore, the expression of p-mTOR/mTOR in the model group was significantly higher than that in the C group (*P* < 0.001). The p-AMPK/AMPK expression in the metformin + rapamycin group was significantly higher than that in the model group, and p-mTOR/mTOR expression in the metformin, rapamycin, and metformin + rapamycin groups was significantly lower than that in the model group (*P* < 0.001) (Fig. 3B). The protein expression levels of the autophagy markers LC3II/I and Beclin1 in the model group were significantly lower than those in the C group (P < 0.001). however, treatment with metformin, rapamycin, or metformin + rapamycin increased the LC3II/I and Beclin1 expression levels (Fig. 3C). Furthermore, we also detected the expression of the autophagy markers (ATG5, ATG7 and ATG12), the protein levels of ATG5, ATG7 and ATG12 in the IMN model group were significantly lower than those in the Control group (*P* < 0.001). Treatment with metformin, rapamycin, or metformin + rapamycin increased the ATG5, ATG7 and ATG12 levels (*P* < 0.05, *P* < 0.01, *P* < 0.001), suggesting that metformin combined with rapamycin promoted autophagy (Fig. 3D).

### Metformin combined with rapamycin impeded the inflammatory response and promoted autophagy in podocytes

**Fig. 4:**
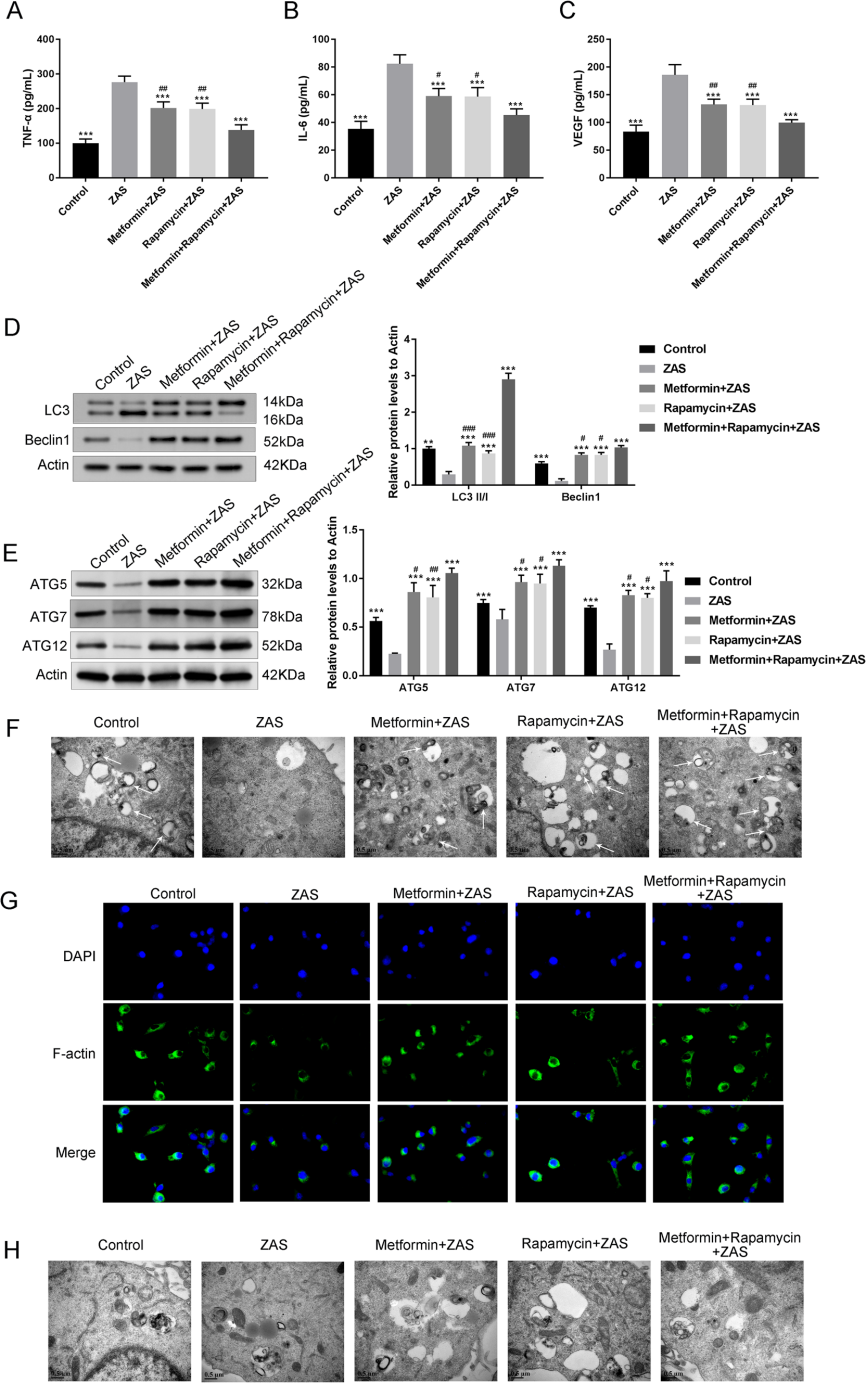
Metformin combined with rapamycin impeded the inflammatory response and promoted autophagy in podocytes. **A-C** The levels of TNF-a (**A**), IL-6 (**B**), and VEGF (**C**) in the MPC-5 cell supernatant measured using ELISA. **D** Western blot analysis of the protein expression levels of autophagy markers (LC3I, LC3II, and Beclin1) in MPC-5 cells. **E** Western blot analysis of the protein expression levels of autophagy markers (ATG5, ATG7, and ATG12) in MPC-5 cells. **F** Representative autophagic vacuoles in MPC-5 podocytes observed using transmission electron micrographs (TEM) (Scale bars = 0.5 μm). **G** The damage degree of the cytoskeletal protein F-actin in MPC-5 podocytes detected using immunofluorescence. **H** Cytoskeleton integrity in each group of MPC-5 podocytes observed using TEM (scale bars = 0.5 μm). **P < 0.01, ***P < 0.001 vs. zymosan-activated serum (ZAS) group; ^#^*P* < 0.05, ^##^*P* < 0.01, ^###^*P* < 0.001 vs. metformin + rapamycin + ZAS group

A medium with 10% ZAS was used to treat MPC-5, as zymosan promotes rapid C3 cleavage through a different pathway to form C5b-9. The concentrations of TNF-α, VEGF, and IL-6 in the MPC-5 supernatant of each group were determined using ELISA (Fig. 4A-C). TNF-α, IL-6, and VEGF levels increased after podocyte exposure to ZAS. However, metformin, rapamycin, and metformin + rapamycin decreased the levels of these factors in MPC-5 after exposure to ZAS (*P* < 0.001). Metformin + rapamycin showed a relatively enhanced effect compared with that of metformin or rapamycin treatment alone. Moreover, the levels of autophagy markers (LC3I, LC3II, and Beclin1), ATG5, ATG7, ATG12 were assessed in the podocyte sublytic C5b-9 membrane attack mode, and it was observed that ZAS induced a significant decrease in the expression levels of LC3II/I, Beclin1, ATG5, ATG7, ATG12 (*P* < 0.001). In addition, metformin, rapamycin, and metformin + rapamycin restored the expression of LC3II/I, Beclin1, ATG5, ATG7, ATG12 in MPC-5 cells after exposure to ZAS (*P* < 0.05, *P* < 0.01, Fig. 4DE). Autophagic vacuoles were examined using TEM. ZAS treatment induced a significant decrease in the number of autophagic vacuoles (Fig. 4F). Actin reorganization was considered a marker of podocyte foot process disappearance in an in vitro study. After exposure to ZAS, the staining of F-actin stress fibers significantly decreased in podocytes, and metformin, rapamycin, and metformin + rapamycin increased the number of F-actin stress fibers in MPC-5 after exposure to ZAS (Fig. 4G). TEM showed the same results for the degree of damage of the podocyte cytoskeleton, suggesting that metformin, rapamycin, and metformin + rapamycin treatments could improve the cytoskeleton structure, as demonstrated by the more orderly arrangement and preserved cell polarity (Fig. 4H).

## Discussion

IMN can cause adult nephrotic syndrome, and advanced drugs are needed for its treatment. In the present study, the effects of metformin and rapamycin combination treatment on C-BSA-induced IMN model rats and their possible mechanisms were investigated.

IMN is characterized by the development of subepithelial immune complex deposits that invade podocytes and demonstrate proteinuria during this process (Tian et al. 2019). C-BSA-induced nephropathy simulates IMN pathological manifestations and is extensively utilized in human IMN studies, providing a useful tool for studying mechanisms and drug screening (Border et al. 1982). Thus, we used C-BSA to establish a rat model of IMN and found that IMN rats had increased 24-h urinary protein, BUN, and serum creatinine levels. According to pathological analysis, the kidney tissues of IMN rats demonstrated slightly increased glomeruli and irregularly thickened capillary walls. According to our data, metformin + rapamycin treatment reduced proteinuria and glomerular pathomorphology in C-BSA-treated IMN rats. Metformin and rapamycin have been shown to have anti-inflammatory effects (Han et al. 2018; Ma et al. 2018; Na et al. 2021; Wen et al. 2019). At present, metformin combined with rapamycin treatment reduces the inflammatory response in model IMN rats.

Although the mechanisms underlying MN remain unclear, podocyte injury can play a major role in MN occurrence (Lai et al. 2009; Meyer-Schwesinger et al. 2020; Tian et al. 2019). The AMPK-mTOR signaling pathway is a typical autophagy regulatory signaling pathway (Simon et al. 2017; Yao et al. 2020). Furthermore, emodin has been found to suppress cell apoptosis and improve podocyte autophagy through the AMPK/mTOR signaling pathway in the renal organs of rats with diabetic nephropathy (Liu et al. 2021). Autophagy is increased by AMPK and inhibited by mTOR. It has been reported that rapamycin is a selective mTOR inhibitor (Zhou et al. 2017). Furthermore, metformin exhibits an antitumor effect through the AMPK/mTOR signaling pathway (Yue et al. 2014). In this study, podocytes maintained a certain degree of autophagy under normal conditions. A few autophagosomes were also observed in group C. A recent study reported that insufficient autophagy was detected in podocytes and that excessive proteinuria was accompanied by podocyte loss (Dong et al. 2019). Autophagosomes are absent in the MN model. After treatment with metformin and rapamycin, the presence of autophagosomes slowly increased, implying that autophagy protects against podocyte damage. We studied the expression of autophagy markers LC3-II/I and Beclin-1. In addition, the transformation of LC3-I to LC3-II is considered a marker of autophagy (Tanida et al. 2004). Compared with the C group, the expression levels of LC3-II/I and Beclin-1 were significantly reduced in IMN rats and then restored after the metformin and rapamycin intervention in this study.

The sublytic C5b-9 injury did induce apoptosis in cultured rat and mouse podocyte (Liu et al. 2017; Nauta et al. 2002). We explored the treatment of metformin and rapamycin in MPC-5 podocytes attacked by the sublytic C5b-9 membrane. We found that metformin, rapamycin, and metformin + rapamycin decreased the levels of TNF-α, VEGF, and IL-6 factors and promoted autophagy in MPC-5 after exposure to ZAS. Furthermore, we observed that metformin and rapamycin could improve the cytoskeleton structure of MPC-5. These findings were consistent with our in vivo results. Autophagy can remove damaged proteins and degrade organelles to maintain podocyte function (Mizushima and Komatsu 2011). Metformin combined with rapamycin can positively regulate the autophagic process in IMN rats and MPC-5 podocytes through the AMPK/mTOR signaling pathway.

Here, metformin combined with rapamycin ameliorated podocyte injury in IMN through the promotion of autophagy via the AMPK/mTOR signaling pathway, suggesting that the combined use of metformin and rapamycin might serve as a novel treatment strategy against IMN.

### Supplementary Information

Below is the link to the electronic supplementary material.

The kidney samples were removed from experimental rats of each group
