# Supplementary figures and images for: Metformin combined with rapamycin ameliorates podocyte injury in idiopathic membranous nephropathy through the AMPK/mTOR signaling pathway
Source: J Cell Commun Signal. 2023 Sep 13;17(4):1405–15. doi: 10.1007/s12079-023-00781-8 (PMC10713903; doi:10.1007/s12079-023-00781-8)

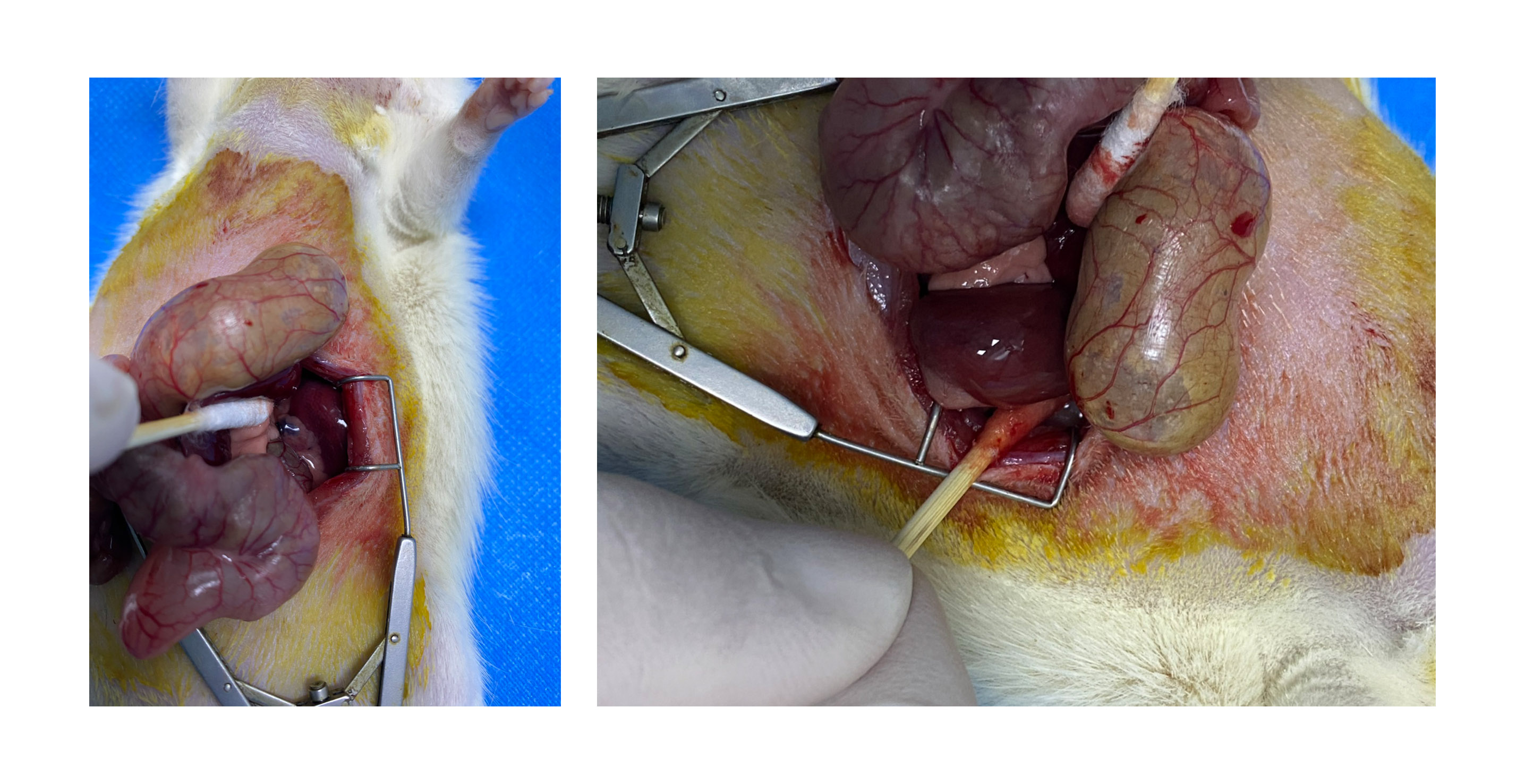

Supplement: Supplementary file 1 — The kidney samples were removed from experimental rats of each group [file 12079_2023_781_MOESM1_ESM.jpg]
